# A Novel Six-Gene-Based Prognostic Model Predicts Survival and Clinical Risk Score for Gastric Cancer

**DOI:** 10.3389/fgene.2021.615834

**Published:** 2021-02-22

**Authors:** Juan Li, Ke Pu, Chunmei Li, Yuping Wang, Yongning Zhou

**Affiliations:** ^1^Department of Gastroenterology, The First Hospital of Lanzhou University, Lanzhou, China; ^2^Key Laboratory for Gastrointestinal Diseases of Gansu Province, The First Hospital of Lanzhou University, Lanzhou, China; ^3^Department of Gastroenterology, Gansu Provincial Hospital, Lanzhou, China; ^4^Department of Oncology, The First Hospital of Lanzhou University, Lanzhou, China

**Keywords:** gastric cancer, autophagy-related genes, overall survival rate, risk-score model, biomarkers

## Abstract

**Background:** Autophagy plays a vital role in cancer initiation, malignant progression, and resistance to treatment. However, autophagy-related genes (ARGs) have rarely been analyzed in gastric cancer (GC). The purpose of this study was to analyze ARGs in GC using bioinformatic analysis and to identify new biomarkers for predicting the overall survival (OS) of patients with GC.

**Methods:** The gene expression profiles and clinical data of patients with GC were obtained from The Cancer Genome Atlas (TCGA) and Gene Expression Omnibus (GEO) datasets, and ARGs were obtained from two other datasets (the Human Autophagy Database and Molecular Signatures Database). Lasso, univariate, and multivariate Cox regression analyses were performed to identify the OS-related ARGs. Finally, a six-ARG model was identified as a prognostic indicator using the risk-score model, and survival and prognostic performance were analyzed based on the Kaplan-Meier test and ROC curve. Estimate calculations were used to assess the immune status of this model, and Gene Ontology (GO) and Kyoto Encyclopedia of Genes and Genomes (KEGG) analyses were employed for investigating the functions and terms associated with the model-related genes in GC.

**Results:** The six ARGs, *DYNLL1*, *PGK2*, *HPR*, *PLOD2*, *PHYHIP*, and *CXCR4*, were identified using Lasso and Cox regression analyses. Survival analysis revealed that the OS of GC patients in the high-risk group was significantly lower than that of the low-risk group (*p* < 0.05). The ROC curves revealed that the risk score model exhibited better prognostic performance with respect to OS. Multivariate Cox regression analysis indicated that the model was an independent predictor of OS and was not affected by most of the clinical traits (*p* < 0.05). The model-related genes were associated with immune suppression and several biological process terms, such as extracellular structure organization and matrix organization. Moreover, the genes were associated with the P13K-Akt signaling pathway, focal adhesion, and MAPK signaling pathway.

**Conclusions:** This study presents potential prognostic biomarkers for GC patients that would aid in determining the best patient-specific course of treatment.

## Introduction

Gastric cancer (GC) is a global health problem. More than one million people are newly diagnosed with GC every year, making it the fifth most commonly diagnosed malignancy worldwide. Moreover, the fact that GC is usually at an advanced at the time of diagnosis results in a high mortality rate. It is the third most common cause of cancer-associated death, after lung and colorectal cancers, with 784,000 deaths registered globally in 2008 (Bray et al., 2018). East Asia, including China, Japan, and South Korea is home to half of all the newly diagnosed cases (Ajani et al., 2016; Chen et al., 2016; Nomura et al., 2017). Meanwhile, the incidence of GC is two times higher in men than that in women (World Health Organization, 2020).

At present, the main treatment options for GC include surgery and chemoradiotherapy (McLean and El-Omar, 2014). Although developments in chemotherapy have reduced mortality in patients with GC, it remains a major global public health challenge, with a 5-year survival rate of <10% (Orditura et al., 2014).

Autophagy, the phenomenon of cell self-digestion, was first proposed by de Duve in 1963 (Galluzzi et al., 2017). The lysosomal degradation pathway involved in autophagy plays a fundamental role in cell, tissue, and organism homeostasis. This highly conserved multi-step catabolic pathway is mediated by evolutionarily conserved autophagy-related genes (ARGs; Ricci, 2016; Budini et al., 2017). The autophagy pathway can be broadly categorized into three major types, i.e., macroautophagy, microautophagy, and chaperone-mediated autophagy (Jacob et al., 2017). In the context of the survival of normal and tumor cells, autophagy plays contrasting roles. On the one hand, autophagy results in the degradation of dysfunctional proteins and organelles, thus, preventing the accumulation of unnecessary products and inhibiting tissue damage while maintaining host defense (Liang and Jung, 2010). On the other hand, autophagy is believed to have a carcinogenic effect and promote tumor progression. Indeed, autophagy has been associated with resistance to chemotherapy in various tumors (Maes et al., 2013). In recent years, the relationship between autophagy and GC has become a dominant focus of research. Autophagy-related molecules have clinical value and can be used as prognostic markers for GC.

In the present study, we screened autophagy genes related to GC prognosis using bioinformatic tools and established risk score models. The predictive value of different models involving autophagy genes and the overall survival (OS) of patients with GC was then evaluated using data in The Cancer Genome Atlas (TCGA) database. These results were then verified using data in the Gene Expression Omnibus (GEO) database. Finally, GO and Kyoto Encyclopedia of Genes and Genomes (KEGG) analyses were used to investigate the function of autophagy genes and the molecular pathways associated with these genes. In this study, we identified potential prognostic biomarkers that will help clinicians make appropriate treatment decisions.

## Materials and Methods

### Sample and Data Collection

The GDC dataset containing the gene expression profiles (HTSeq-FPKM) of 375 GC patients was downloaded from TCGA-STAD.^1^ To ensure uniformity across data, the RNA-seq profiles were transformed into TPM, and the formula log2(TPM+1) was used for normalization. The clinical, phenotype, and survival data of patients, including age, sex, tumor size, node metastasis, distant metastasis (TNM) stage, tumor grade, and OS time, were obtained using the Xena Browser. After excluding the data of patients with an OS of <30days, the data of 339 patients with GC were used for further analysis. The gene expression profiles and clinical data of 300 patients with GC were obtained from the GEO microarray dataset (GSE62254) as the validation cohort data. The data were analyzed using R.^2^

### Acquisition of a Human Autophagy-Related Gene Set

A total of 232 and 394 ARGs were independently obtained from two datasets of the Human Autophagy Database (HADb)^3^ and the GO enrichment-related autophagy genes (GO_AUTOPHAGY) Molecular Signatures Database (MSigDB v6.2^4^), respectively. After removing the duplicated ARGs from the two datasets, 531 ARGs were included for further analysis.

### Identification of Prognostic ARGs

Initially, univariate Cox regression analysis was performed on TCGA transcriptome data to identify a correlation between the ARGs and OS. Lasso regression analysis was used to improve the performance parameters and decrease the false positives in variables due to overfitting. Multivariate Cox regression analysis was performed to generate OS prognostic risk models using the stepwise regression method to eliminate ARGs that were not significantly associated with OS.

### Construction of a Risk Score Model

Prognosis-related ARGs were selected based on the results of multivariate Cox regression analysis. Then, the risk score model of each patient was calculated using the following formula:

$$\mathrm{risk score}=\sum_{i=1}^{n}\left({\mathrm{coef}}_{i}*{\mathrm{expr}}_{i}\right)$$

in which the variables coef*_i_* and expr*_i_* represent the multivariate Cox regression coefficients and the corresponding expression of individual ARGs, respectively. The median risk score of the patients was regarded as the cutoff point; thus, the patients with GC were divided into high- and low-risk groups.

The prognostic differences in OS between the two groups were analyzed using the Kaplan-Meier curve and log-rank test. Cox regression analysis was used to estimate the predictive role of the risk scores for the clinical traits of patients with GC. The time-dependent ROC curve was used to assess the accuracy of model predictions.

### Immune Infiltration

The R package ESTIMATE was used to detect the status of stromal and immune cells exhibiting the gene expression signatures of interest in malignant tumors. The immune score, stromal score, and ESTIMATE score for all patients whose data was included in TCGA datasets were calculated using the ESTIMATE R package.

### Functional Annotation of ARGs

To investigate the potential tumor-related molecular mechanisms of ARGs, the correlation between gene expression and risk scores was estimated using Pearson’s correlation test. Significant genes were screened out according to the correlation coefficient |*R*| > 0.4 and *p* < 0.05. All genes identified as being significantly correlated with OS were subjected to GO and KEGG pathway enrichment analyses using the R package “clusterProfiler.” *p* < 0.05 and false discovery rate <0.05 were set as the cutoff criteria to screen the annotation information.

### Statistical Analysis

R (https://www.r-project.org/) was used as the main tool for data analysis and mapping; *p* < 0.05 was regarded as significant. The distribution of differences among the variables was then assessed using the chi-squared test or Fisher’s exact test. OS was analyzed using Kaplan-Meier survival curve analysis and the log-rank test. The Cox regression model was used to analyze factors that affected the survival of patients with GC. Univariate and multivariate analyses were also performed using the Cox proportional hazards regression model. Time-dependent ROC analysis was used to evaluate the accuracy of the models that predicted prognosis. ROC curve analysis was also used to estimate the diagnostic value of gene expression. An area under the curve (AUC) value of ≥ 0.75 was considered significant, and values ≥ 0.6 were considered acceptable for predictions.

## Results

### Identification of Prognosis-Related ARGs in GC Tissue Samples

**Figure 1:**
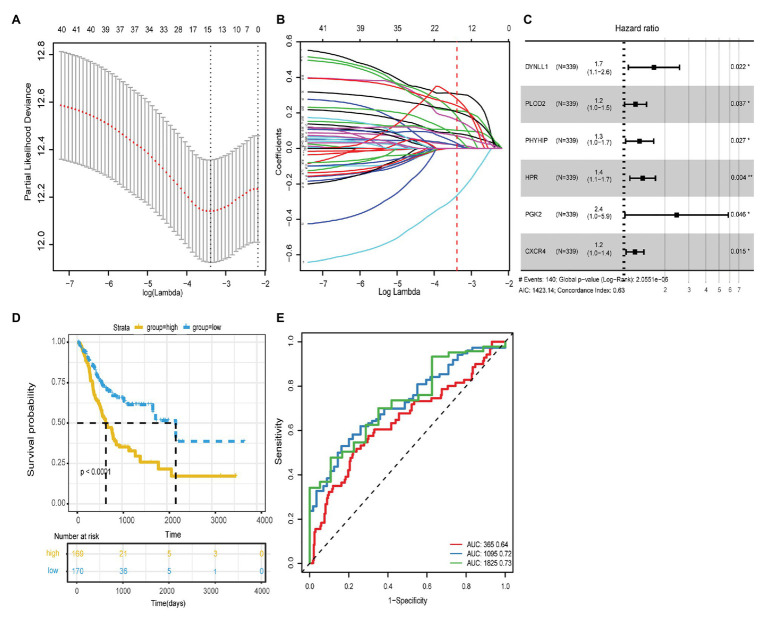
**(A)** The regression analysis of 1,000 times was carried out with Lasso regression, and the single factor appeared more than 500 times was screened. Finally, 15 autophagy genes were obtained. **(B)** Lasso filters variables. **(C)** Forest plot of HR of six genes. **(D)** The ROC curves of risk score model for the prognostic accuracy of GC patients in 1, 3, and 5-year; AUC is the area under the curve, and the larger the value, the better the model. **(E)** Kaplan-Meier survival curve analysis of risk score model.

Based on the transcriptome data in TCGA-STAD, 531 ARGs from HADb (*n* = 232) and MSigDB (*n* = 394) were included in the univariate Cox regression analysis and were screened for GC prognosis. Forty-two ARGs closely correlated with the OS of GC patients were identified (*p* < 0.05; Table 1). Next, Lasso regression analysis was used to eliminate genes that were highly correlated with other genes. Fifteen of the forty-two ARGs that occurred more than 500 times were selected after 1,000-times regression analysis (Figures 1A,B). Multivariate Cox regression was used to analyze the correlation of these 15 genes with GC prognosis. Finally, six genes (*DYNLL1*, *PLOD2*, *PHYHIP*, *HPR*, *PGK2*, and *CXCR4*) significantly associated with GC prognosis were identified using the stepwise regression method (Table 2, Figure 1C). As shown in Figure 1C, all genes that were positively correlated with GC prognosis were identified to be high-risk factors. Importantly, GC patients with high *PGK2* expression had 2.41-times higher mortality risk than patients with low *PGK2* expression (HR: 2.4L, 95% CI 1.0–5.9), indicating that *PGK2* was the most effective prognostic marker among all the analyzed ARGs.

**Table 1:** We performed univariate Cox regression analysis with 531 autophagy-related genes (ARGs) based on TCGA-STAD transcriptome data, and identified 42 ARGs that were associated with the overall survival (OS) of gastric cancer (GC) patients (*p* < 0.05).

Var	HR	CI95·low	CI95·high	*Z* score	*p* value
IRGM	2.664	1.403	5.056	2.996	0.002734
KCNQ1	0.862	0.772	0.962	−2.643	0.008216
STOM	1.231	1.03	1.472	2.281	0.022577
GABARAPL1	1.233	1.012	1.501	2.083	0.037278
CDC37	0.655	0.468	0.916	−2.476	0.013289
HCAR1	1.186	1.037	1.355	2.489	0.012795
PDK4	1.118	1.023	1.221	2.469	0.013565
RRAGD	1.204	1.019	1.423	2.18	0.02923
DYNLL1	1.51	1.011	2.254	2.014	0.044054
CD93	1.208	1.023	1.426	2.229	0.025809
CHMP4C	0.848	0.73	0.985	−2.159	0.030871
BOC	1.145	1.024	1.28	2.377	0.017441
PLOD2	1.34	1.122	1.6	3.229	0.001243
C1orf210	0.844	0.72	0.988	−2.103	0.03544
ANXA5	1.515	1.188	1.931	3.355	7.94e-4
SRPX	1.135	1.017	1.267	2.263	0.023648
MAP1LC3C	1.374	1.036	1.821	2.204	0.027526
PHYHIP	1.409	1.134	1.75	3.096	0.001961
PRKG1	1.185	1.012	1.389	2.107	0.035158
SERPINB10	1.722	1.192	2.486	2.898	0.003757
GABARAPL2	1.986	1.294	3.049	3.137	0.001704
MAP3K12	1.322	1.027	1.702	2.169	0.030051
CPA3	1.125	1.013	1.251	2.192	0.028346
ATG4D	0.75	0.577	0.974	−2.155	0.031178
HPR	1.392	1.137	1.705	3.204	0.001354
FGF7	1.154	1.029	1.293	2.454	0.014131
MAP1LC3B	1.458	1.043	2.038	2.209	0.027205
C5	1.207	1.005	1.449	2.017	0.043656
SFRP4	1.08	1.006	1.159	2.111	0.034734
LENG9	0.802	0.657	0.978	−2.18	0.029247
STK32A	1.488	1.113	1.989	2.686	0.007232
PGK2	2.633	1.191	5.823	2.391	0.016795
PPY	1.353	1.026	1.785	2.138	0.032505
BNIP3	1.162	1.01	1.336	2.106	0.035221
BNIP3L	1.299	1.019	1.658	2.108	0.035059
CXCR4	1.242	1.085	1.422	3.15	0.001633
DLC1	1.25	1.058	1.477	2.623	0.008717
GRID2	1.838	1.055	3.2	2.15	0.031562
HSPB8	1.105	1.012	1.206	2.228	0.025911
MBTPS2	1.315	1.002	1.727	1.973	0.048542
NRG2	1.309	1.029	1.664	2.193	0.028323
NRG3	1.381	1.058	1.803	2.371	0.01773

**Table 2:** The HR of six genes.

Var	HR	CI95.low	CI95.High	*p* value
DYNLL1	1.66	1.08	2.57	0.022
PLOD2	1.22	1.01	1.47	0.037
PHYHIP	1.31	1.03	1.66	0.027
HPR	1.37	1.11	1.71	0.004
PGK2	2.44	1.02	5.86	0.046
CXCR4	1.21	1.04	1.4	0.015

*Variate: the name of the genes; HR: Hazard Ratio; CI95%: 95% confidence interval of HR; p value: p value of survival significance test, <0.05 is significant*.

### Constructing the Prognostic Risk Score Model

Based on the expression levels and multivariate Cox regression coefficients of these six ARGs, the risk scores for individual models were calculated for each patient. The risk score for OS = (0.1504 × expression value of *DYNLL1*) + (0.1673 × expression value of *PLOD2*) + (0.0944 × expression value of *PHYHIP*) + (0.1141 × expression value of *HPR*) + (0.0727 × expression value of *PGK2*) + (0.0103 × expression value of *CXCR4*). Patients with GC were classified into two groups, high-risk (*n* = 170) and low-risk (*n* = 169), according to the cutoff value of the median risk score. The survival status and prognostic ability of the risk score model were evaluated using the Kaplan-Meier curve and area under the ROC curve. The log-rank test revealed that patients with a high-risk score had a poorer prognosis than those with a low-risk score (identified using the Kaplan-Meier curve; *p* < 0.0001; Figure 1D). The area under the ROC curve values were 0.64, 0.72, and 0.73, respectively, for the OS status of 1, 3, and 5years, which indicated that our prognostic models for 3 and 5years had better predictive performance than those for 1year (Figure 1E). Additionally, the other survival-related variables of DFI, DFS, and DSS were discussed, and the log-rank tests also indicated that patients with a high-risk score had worse prognosis compared with those with a low-risk score (Supplementary Figure 1).

**Figure 2:**
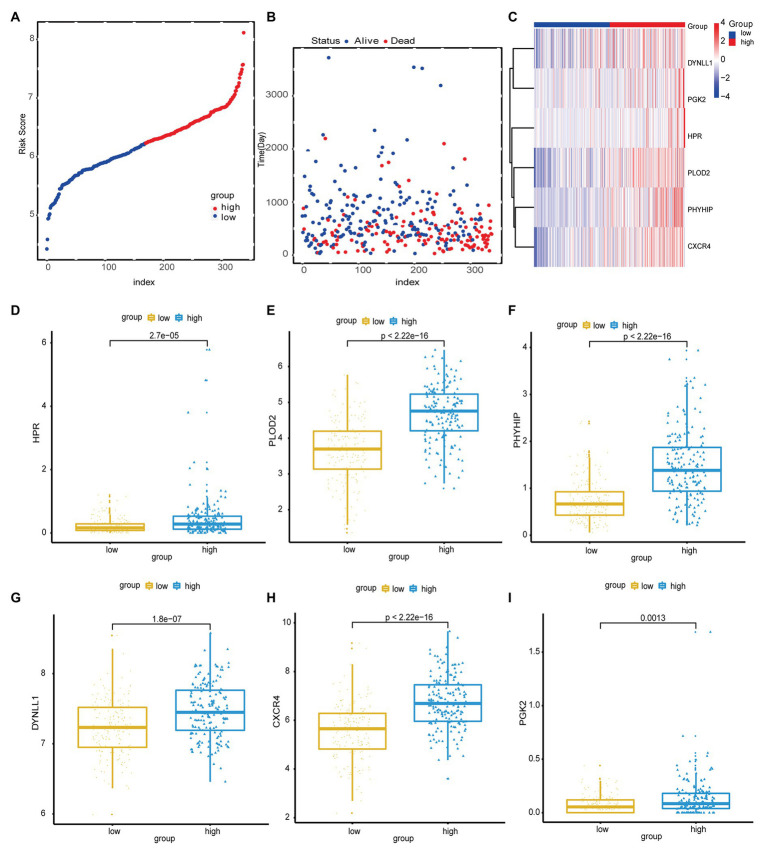
**(A)** Risk score distribution of high-risk group and low-risk group. **(B)** Scatter plot shows the survival status of GC patients with the increasing risk score. **(C)** Heat map shows the expression of autophagy-related genes (ARG) in the high- and low-risk score groups. **(D-I)** The boxplot displayed the expression of HPR, PLOD2, PHYHIP, DYNLL1, CXCR4, and PGK2 in the high- and low-risk score groups.

Next, the risk scores for OS were ranked (Figure 2A); their distribution is shown in Figures 2A,B. Dot plots revealed the OS status of individual patients with GC (Figure 2B). The OS of the majority of patients with GC was distributed over 1,000days (3years). A heatmap was used to display the expression pattern of risk genes in the high- and low-risk groups (Figure 2C). As shown in Figure 2C, patients with high-risk scores in the model exhibited upregulation of *CXCR4*, *PLOD2*, *HPR*, *DYNLL1*, *PGK2*, and *PHYHIP*, in contrast to patients with low-risk scores. Meanwhile, significant differences were observed in the expression of all six ARGs between groups (high- vs. low-risk scores; *p* < 0.05; Figures 2D-I).

### Association Between the Risk Signature and Clinical Characteristics

**Figure 3:**
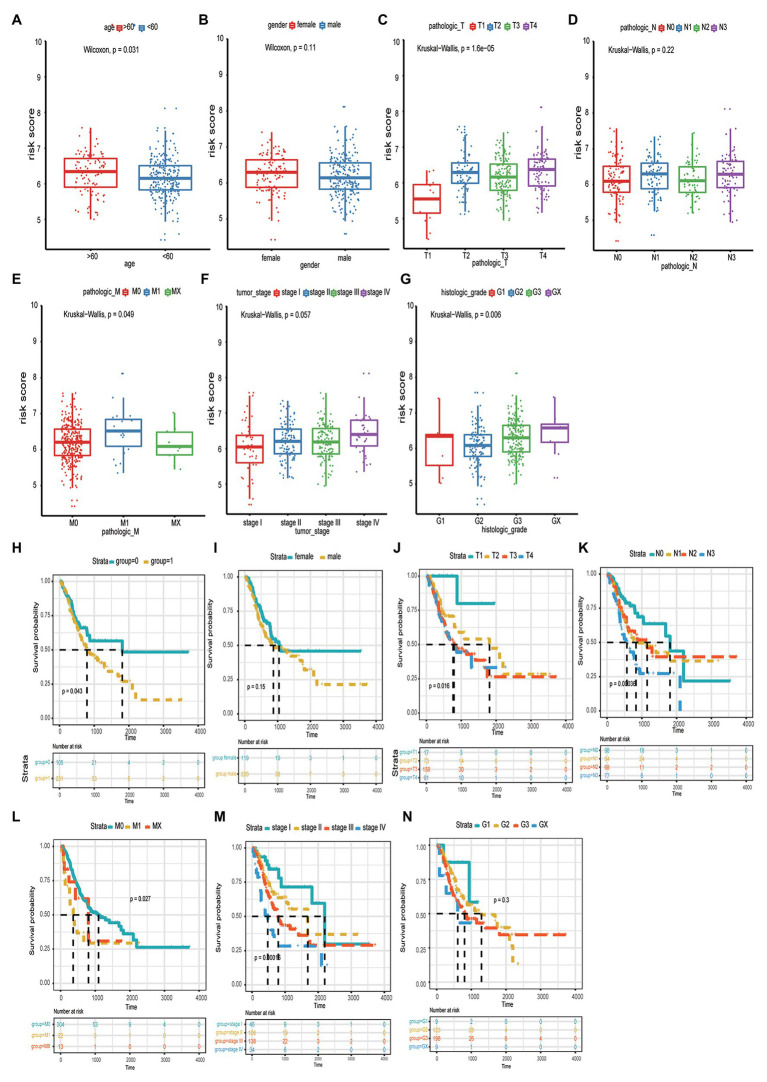
The correlation between risk score and age **(A)**, gender **(B)**, tumor size **(C)**, lymphatic node metastasis **(D)**, distant organ metastasis **(E)**, clinical-stage **(F),** and histologic grade **(G)**. The distribution of the survival curves for different clinical traits, including age **(H)**, gender **(I)**, pathological tumor size, node metastasis, distant metastasis (TNM) stage **(J-L)**, clinical-stage **(M),** and pathological grade **(N)**.

We analyzed the distribution of risk scores for different clinical traits, including age, sex, pathological stage, TNM stage, and tumor grade. As shown in Table 3 and Figures 3A-G, patients with a high-risk score tended to include those who were older (>60years; *p* < 0.031), had a greater tumor width and depth (*p* < 0.000), distant metastases (*p* < 0.049), and a highly invasive histological grade (*p* < 0.006), compared to those with low-risk scores. Additionally, we analyzed the distribution of the survival curves for different clinical traits, including age, sex, pathological stage, TNM stage, and tumor grade (Figures 3H-N). The Kaplan-Meier test revealed that patient age, tumor size, distant metastases, and histological grade were closely associated with patient prognosis.

**Table 3:** Clinical information of the two groups.

	Level	High	Low	*p* value
		*N* = 170	*N* = 169	
Age (median [IQR])		64.50 [56.25, 71.75]	69.00 [60.00, 75.00]	0.003
Gender (%)	FEMALE	69 (40.6)	50 (29.6)	0.04
	MALE	101 (59.4)	119 (70.4)	
Pathologic_T (%)	T1	2 (1.2)	15 (8.9)	0.002
	T2	38 (22.4)	35 (20.7)	
	T3	75 (44.1)	83 (49.1)	
	T4	55 (32.4)	36 (21.3)	
Pathologic_N (%)	N0	44 (26.0)	54 (32.1)	0.164
	N1	52 (30.8)	42 (25.0)	
	N2	29 (17.2)	39 (23.2)	
	N3	44 (26.0)	33 (19.6)	
Pathologic_M (%)	M0	150 (88.2)	154 (91.1)	0.046
	M1	16 (9.4)	6 (3.6)	
	MX	4 (2.4)	9 (5.3)	
Tumor_stage (%)	stage I	18 (11.2)	28 (17.1)	0.089
	stage II	52 (32.5)	54 (32.9)	
	stage III	67 (41.9)	71 (43.3)	
	stage IV	23 (14.4)	11 (6.7)	
Histologic_grade (%)	G1	5 (2.9)	4 (2.4)	0.036
	G2	49 (28.8)	74 (43.8)	
	G3	111 (65.3)	87 (51.5)	
	GX	5 (2.9)	4 (2.4)	

**Figure 4:**
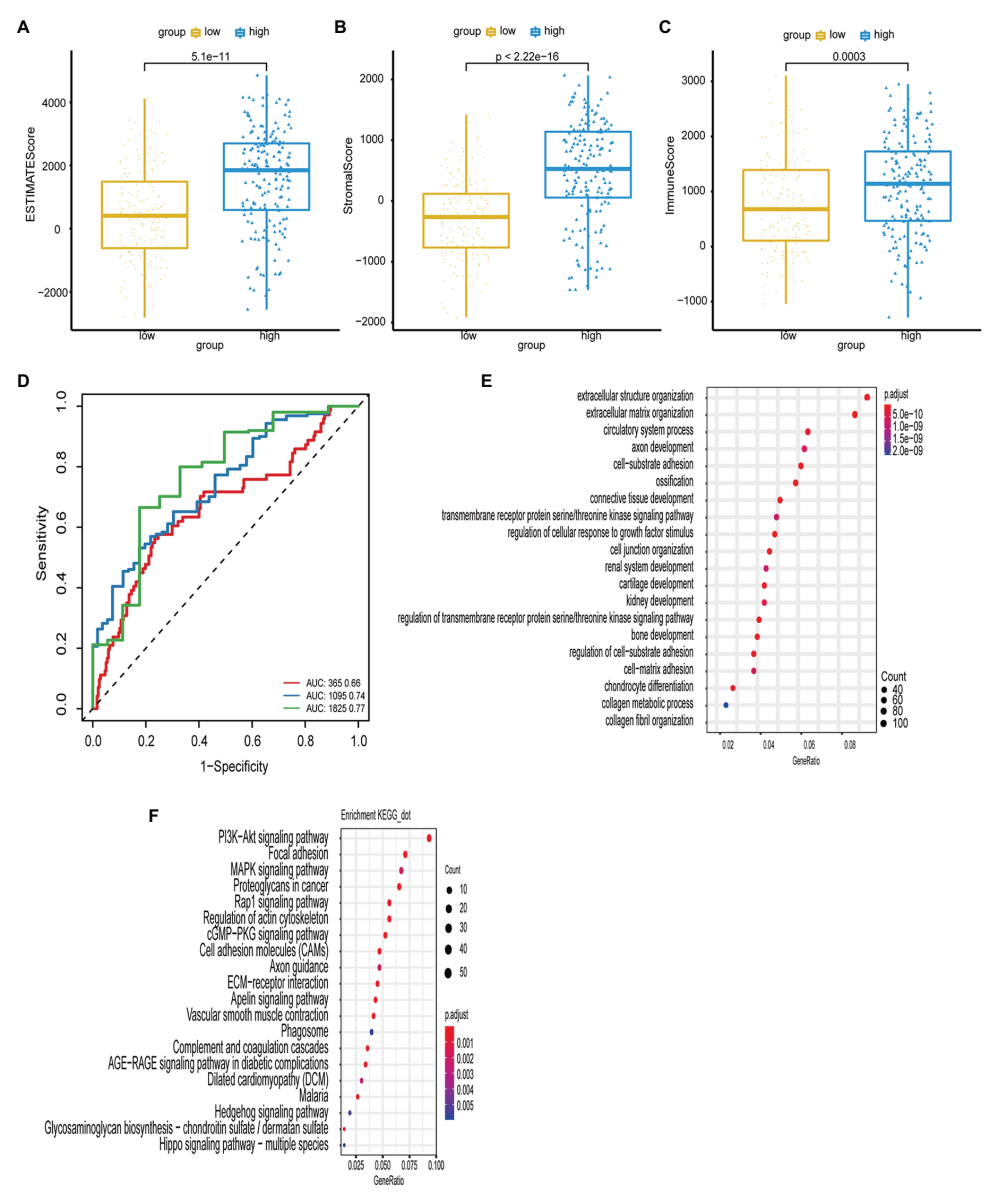
**(A,B,C)** The boxplot shows the infiltration distribution status of GC including ESTIMATE score, stromal score, immune score in the two groups of risk score. The immune scores between groups were tested by *t*-test. **(D)** The ROC curves of risk score model and multiple clinical traits (age, sex, TNM stage, clinical stage, and histological grade) for the prognostic accuracy of GC patients in 1, 3, and 5-year. **(E,F)** The bubble chart shows the result of GO and KEGG enrichment analysis of ARGs.

Moreover, the correlation between the risk score model and OS was analyzed using univariate and multivariate Cox regression analyses. Univariate Cox regression analysis revealed that patient age, pathological stage, tumor invasive size, lymph node metastases, distant metastases, and risk score model were significantly associated with GC patient prognosis. However, after controlling for the confounding factors, multivariate Cox regression analysis revealed that age and the risk score model were independently correlated with OS (Table 4). These results indicated that the risk score model could be regarded as an independent predictor of GC prognosis. Moreover, based on the risk score and clinical traits, we further evaluated the predictive performance of the model with respect to OS of 1, 3, and 5years, respectively (ROC curve analysis; Figure 4D). The area under the ROC curve values of 0.74 and 0.77 in 3- and 5-year OS of patients with GC, respectively, demonstrated that the risk score model had a higher predictive performance for GC prognosis.

**Table 4:** Cox regression analysis of risk score and clinical traits in TCGA dateset.

Var	HR	CI95.low	CI95.high	Z_score	P value	M_HR	M_CI95.low	M_CI95.High	M_Z score	M_P value
Gender	1.301	0.908	1.864	1.436	0.151131	NA	NA	NA	NA	NA
Age	1.477	1.01	2.161	2.009	0.044516	1.77	1.19	2.63	2.813	0.004914
Tumor stage	1.931	1.34	2.784	3.529	4.18e-4	1.37	0.82	2.29	1.196	0.231624
Histologicgrade	1.354	0.955	1.918	1.703	0.088575	NA	NA	NA	NA	NA
Pathologic_T	1.79	1.171	2.735	2.688	0.007185	1.17	0.71	1.93	0.607	0.544117
Pathologic_N	1.983	1.295	3.037	3.148	0.001643	1.36	0.78	2.38	1.089	0.276272
Pathologic_M	1.805	1.099	2.963	2.334	0.019592	1.41	0.82	2.42	1.24	0.214922
Risk_score	2.718	1.974	3.742	6.131	0	2.63	1.89	3.65	5.765	0

*Variate: clinical traits; HR (M_HR): Hazard Ratio values in univariate (multivariate) Cox regression analysis; CI95% (M_CI95%): 95% confidence interval of HR in univariate (multivariate) Cox regression analysis; z_score (M_z_score): value of z test in univariate (multivariate) Cox regression analysis; p value (M_P_value): p value of univariate (multivariate) Cox regression analysis, <0.05 is significant*.

### Functional Characteristics of ARGs

To determine the immune status in the high-risk tumors from patients with GC, ESTIMATE analysis was performed for the high- and low-risk groups. As shown in Figures 4A-C, the immune, stromal, and ESTIMATE scores were significantly elevated in the high-risk group compared with those in the low-risk group (*p* < 0.05), suggesting that immune suppression was associated with high-risk tumors.

Furthermore, we used GO and KEGG enrichment analyses to investigate the function of the genes. Pearson’s correlation analysis was performed to identify a correlation between the risk score and all genes from TCGA-STAD (Pearson *R* > 0.4, *p* < 0.05). GO analysis revealed that these genes were associated with a range of biological process terms, including extracellular structure organization, extracellular matrix organization, and circulatory system processes. KEGG pathway enrichment analysis revealed that these genes were particularly associated with the PI3K/Akt signaling pathway, focal adhesion, and MAPK signaling pathway (Figures 4E,F).

### Verification of Risk Score Models for the GEO Dataset

**Figure 5:**
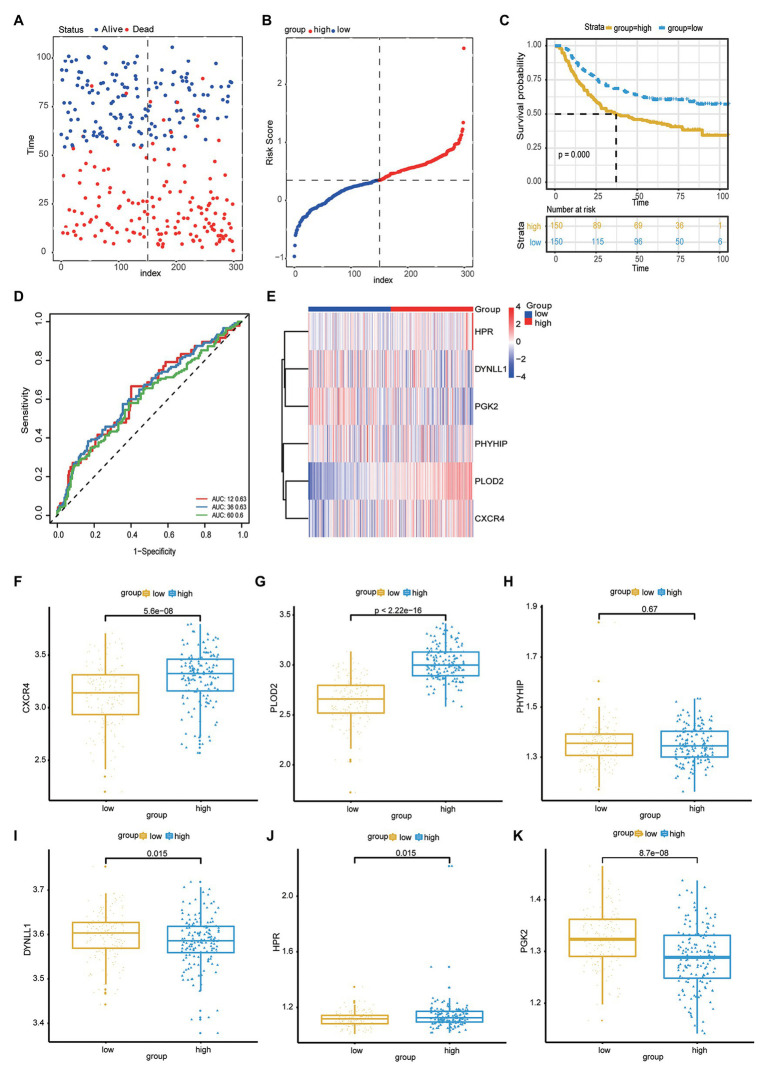
**(A)** Scatter plot shows the survival status of GC patients. **(B)** Risk score distribution of high- and low-risk group. **(C)** The Kaplan-Meier plot of the risk score model to show the for the GC prognostic status. **(D)** The ROC curves of risk score model for the prognostic accuracy of GC patients in one-year, three-years and five-years. **(E)** Heat map shows the expression of autophagy-related genes in the high risk score group and low risk score group. **(F-K)** Boxplot to show the expression of CXCR4, PLOD2, PHYHIP, DYNLL1, HPR, PGK2 in the high- and low-risk group.

The accuracy of the risk score model was validated using the testing set from among the patients within the GC cohort of the GEO datasets. First, the risk score model for each patient with GC was calculated and constructed based on the expression levels of *DYNLL1*, *PLOD2*, *PHYHIP*, *HPR*, *PGK2*, and *CXCR4*, in the GEO microarray data. The patients were subdivided into high- and low-risk groups according to the median risk score of the cohort population. The distributions of risk score and survival status are shown in Figures 5A,B. Furthermore, Kaplan-Meier analysis of the prognostic performance of the risk score model with respect to GC prognosis indicated that the high-risk group was associated with a poor prognosis compared to the low-risk group (Figure 5C). The diagnostic performance of the risk score model – based on ROC curve analysis – revealed that this model predicted moderate OS for patients with GC [AUC values of 1-year (0.63), 3-year (0.63), and 5-year (0.60); Figure 5D]. This indicated that the prediction of GC prognosis was acceptable. Heatmap analysis revealed the expression patterns of risk genes in the high- and low-risk groups (Figure 5E). As shown in the boxplot (Figures 5F-K), the expression of *CXCR4*, *PLOD2*, and *HPR* was significantly upregulated in patients with GC, while the expression of *DYNLL1* and *PGK2* was significantly downregulated in patients with GC in the GEO dataset (*p* < 0.05; Figures 5F-K). Additionally, the association between the risk score model and independent predictive factors was analyzed using univariate and multivariate regression analyses. Variations in patient age, sex, pathological stage, tumor invasive size, lymph node metastases, and distant metastases were included in the regression analysis. After controlling for the confounding factors, multivariate Cox analysis indicated that distant metastases, pathological stage, and the risk score model were independently correlated with OS in patients with GC (Table 5). These results also indicated that the risk score model could be regarded as an independent prognostic marker for GC in the validation set.

**Table 5:** Cox regression analysis of risk score and clinical traits in GEO dateset.

Var	HR	CI95·low	CI95·high	*Z* score	*p* value	M_HR	M_CI95·low	M_CI95·high	M_Z_score	M_P_value
Age	1.239	0.882	1.741	1.235	0.216955	NA	NA	NA	NA	NA
Sex	0.905	0.647	1.265	−0.585	0.558748	NA	NA	NA	NA	NA
Pathologic T	2.396	1.741	3.297	5.362	0	1.26	0.85	1.86	1.161	0.245454
Pathologic N	2.816	1.434	5.527	3.009	0.002625	1.79	0.88	3.68	1.598	0.110025
Pathologic M	3.84	2.482	5.942	6.042	0	2.46	1.56	3.88	3.889	1.01e-4
P Stage	3.472	2.385	5.053	6.498	0	2.17	1.34	3.52	3.134	0.001726
Risk score	2.718	1.749	4.226	4.442	9e-6	2.16	1.34	3.49	3.156	0.001599

*Variate: clinical traits; HR (M_HR): Hazard Ratio values in univariate (multivariate) Cox regression analysis; CI95% (M_CI95%): 95% confidence interval of HR in univariate (multivariate) Cox regression analysis; z_score (M_z_score): value of z test in univariate (multivariate) Cox regression analysis; p value (M_P_value): p value of univariate (multivariate) Cox regression analysis, <0.05 is significant*.

## Discussion

Gastric cancer is a malignant cancer with a high mortality rate. Most patients with GC are diagnosed at the terminal stage of the disease due to its nonspecific clinical symptoms in the early stages, which also creates a challenge for treatment (Craanen et al., 1991; Inoue and Tsugane, 2005; Kim et al., 2015). The early detection of GC could reduce the mortality rate by 30–65% (Hosokawa et al., 2008; Lee et al., 2013; Pasechnikov et al., 2014). Currently, endoscopic biopsy remains the most efficient option for early GC detection and prognostic assessment. Nevertheless, endoscopic tests and biopsies are invasive, unpleasant, and inconvenient, contributing to potential errors for GC detection and prognosis. Therefore, it is vital to identify ideal biomarkers for the early diagnosis and prognosis of GC. Serum-based biomarkers, including CA724, CEA, CA125, and CA199, play an essential role in the early diagnosis and prognosis of GC (Yang et al., 2014; Coghlin and Murray, 2016). Although our understanding of GC biology has grown significantly over the last decade, practical information for the screening and diagnosis of GC remains limited. Moreover, there are no specific biomarkers to accurately diagnose early GC or monitor patient responses to treatment. Inhibition or upregulation of autophagy may modulate the metabolic reprogramming of cancer cells (White et al., 2015; Kimmelman and White, 2017). At present, whether autophagy plays an important role in GC is uncertain. Autophagy may inhibit the initiation of cancer, but a positive association between autophagy and tumor metastasis and various therapeutic responses has also been suggested (Janku et al., 2011; Giuliano et al., 2015).

In the present study, we first analyzed mRNAs from TCGA to identify key ARGs relevant for prognosis in patients with GC. Six ARGs (*DYNLL1, PLOD2, PHYHIP, HPR*, *PGK2*, and *CXCR4*) were found to be significantly associated with OS in GC patients using the Lasso and Cox regression analyses. So we focused on OS in our study. Then, the prognostic risk score model was generated according to the coefficient and expression levels of ARGs in each patient. Survival-related variables (OS, DFI, DFS, and DSS) and prognostic ability analyses indicated that the high-risk score model could distinguish patients with better prognosis. The correlation between OS and risk score was mainly discussed in this study. Furthermore, with respect to the effect of clinical traits on the association between this model and OS, multivariate Cox regression analysis suggested that the risk score model was independently correlated with OS in patients with GC. The high-risk score model of ARGs was associated with an adverse outcome in GC. Subsequently, we analyzed the model-related ARGs using the GEO microarray for the validation cohort. Survival and prognostic ability analyses also showed that the high-risk score model was correlated with adverse outcomes in patients with GC. Although the diagnostic performance of the model decreased compared with the first evaluation, many factors, such as the quality of samples and defeat data from the microarray were involved. Additionally, multivariate Cox regression analysis suggested that the risk score model was an independent predictive factor for the OS in GC.

Recently, several biomarkers for GC prognosis have been identified, including mRNAs, non-coding RNAs, and proteins. A risk score model based on four genes (*GRID2*, *ATG4D*, *GABARAPL2*, and *CXCR4*) was identified as a potential prognostic biomarker for OS of GC (AUC, 0.671; Qiu et al., 2020). Another study suggested a high prognostic accuracy of a four-DNA methylation signature in GC patients (AUC, 0.724; Li et al., 2020). A seven-miRNA biomarker panel (miR-10b, miR-223, miR-30a-5p, miR-126, miR-21, miR-338, and let-7a) was established for OS prediction and validated using an independent dataset (Li et al., 2010). Additionally, long non-coding (lnc) RNA risk score models as prognostic indicators for GC have also been reported in many studies. A nine-lncRNA signature model (AUC, 0.795) had moderate prognostic ability for the 5-year OS of GC (Cai et al., 2019), another study reported its predictive ability for a 5-year OS (AUC, >0.7; Nie et al., 2020). Additionally, some studies have established Her-2 as a prognostic biomarker for GC and to assess the effectiveness of the targeted drugs (Begnami et al., 2011; Gordon et al., 2013).

Although comparisons with mRNA, miRNA, and lncRNA risk score models and protein signatures suggested the superior efficiency of our model with respect to predictive performance, minority of studies discussed the better prognostic models of multiple genes for DFI, DFS, and DSS (Lee et al., 2016; Guan et al., 2020). Meanwhile, large-scale cohort validation was absent for these biomarkers. It was unclear whether these signatures had clinic applicability with high predictive ability.

To further discuss the correlation between the risk score model and immunity status of GC, the high stromal and immune scores distributed in the high-risk group were compared with those of the low-risk group. In fact, the stromal cell ratio of the tumor microenvironment is the predictive indicator of poor prognosis of multiple malignancies (Panayiotou et al., 2015). However, the prognostic effects of GC differed in tumor-associated immune cell infiltration. Various immune cells, including macrophages and T lymphocytes, are involved in GC progression. Among these, macrophage infiltration can increase the invasion and metastasis of GC, whereas T lymphocytes are positively associated with a favorable prognosis of GC by inhibiting tumor progression (Lee et al., 2008; Zheng et al., 2017). In this study, a high-risk score model indicating adverse prognosis was established based on the presence of high tumor-associated stromal and immune cells, but there was no evidence of the potential mechanism between model-related ARGs and stromal and immune cells.

Additionally, the enrichment analyses revealed that the model-associated genes were mainly associated with process terms, such as extracellular structure organization and matrix organization. Moreover, the genes were particularly associated with the PI3K/AKT signaling pathway and focal adhesion. In fact, the PI3K/Akt signaling pathway was activated by multiple stimuli and amplified, mutated, and translocated more frequently than other pathways (Sun et al., 2017). Meanwhile, the PI3K/Akt pathway protected the gastric mucosal epithelium from damage, which is closely correlated with the invasion and metastasis of various malignancies (Zhang et al., 2017).

*DYNLL1* is a protein-coding gene. Among its related pathways are cell cycle_spindle assembly and chromosome separation and Organelle biogenesis and maintenance. *DYNLL1*-related terms include transcription factors, DNA damage response proteins, apoptosis regulators, synaptic transmission, and cell migration (Li and Vederas, 2009; Saito, 2009; Vera et al., 2009). Previous studies have suggested that *DYNLL1* is an important factor that can affect genomic stability and response to DNA-damaging chemotherapy (He et al., 2018). The downregulation of *DYNLL1* is significantly correlated with poor progression-free survival in patients with *BRCA1*-mutated ovarian carcinomas undergoing platinum-based chemotherapy (He and Meghani and Caron and Yang and Ronato and Bian and Sharma and Moore and Niraj and Detappe and Doench and Legube and Root and D’Andrea and Drane and De and Konstantinopoulos and Masson and Chowdhury 2018). Berkel and Cacan (2020) found that hepatocellular carcinoma (HCC) patients with the higher expression level of *DYNLL1* were associated with both shorter OS and shorter progression-free survival and upregulated in a tumor stage- and grade-dependent manner and associated with increased mortality in HCC. In the present study, the expression of *DYNLL1* was upregulated in high-risk groups and downregulated in low-risk groups. These findings were consistent with the high expression of *DYNLL1*, which is indicative of a poor prognosis of GC patients.

*PGK2* codes for an important enzyme in the glycolysis pathway that catalyzes the conversion of glycerol-1, 3-diphosphate into 3-phosphoglycerate (Wu et al., 1997). Increased *PGK2* expression reflects rapid tumor growth and increased growth in anaerobic conditions (Semenza et al., 1994; Semenza, 1999). In the present study, we found that PGK2 was upregulated in high-risk patients with GC. This can be partly explained by the high expression of *PGK2*, which enables the tumor cells to tolerate hypoxia and allows these cells to acquire compounds for synthesis and metabolism through the glycolysis pathway. Consequently, targeting the activity of glycolytic enzymes may be a promising strategy for these patients.

*CXCR4*, as the most common chemokine receptor, is responsible for numerous malignancies, including breast cancer, melanoma, prostate cancer, and GC (Lee et al., 2009; Fanelli et al., 2012). It can regulate epithelial-to-mesenchymal transition through the PI3K/AKT pathway in GC (Gao et al., 2014; Fontanella et al., 2016; Lin et al., 2017; Bao et al., 2019). Meanwhile, high expression of *CXCR4* is associated with lymphatic metastasis, advanced pathological stages, and a poor prognosis for patients with GC. *CXCR4* plays an essential role in vascularization of the gastrointestinal tract, probably by regulating vascular branching and/or remodeling processes in endothelial cells (Gupta and Pillarisetti, 1999). In this model, the upregulation of *CXCR4* in the high-risk groups was also positively correlated with the poor prognosis of patients with GC.

*PLOD2* is located on chromosome 3q23q24 and encodes a membrane-bound homo-dimeric enzyme that hydroxylates lysines in the telopeptide of procollagens (Szpirer et al., 1997; Du et al., 2017), which is involved in extracellular matrix formation and numerous pathological processes in malignancies (Lu et al., 2012; Giussani et al., 2015). Silencing *PLOD2* expression in cancer-associated fibroblasts significantly reduces tumor invasion and metastasis (Pankova et al., 2016). Therefore, *PLOD2* may affect cancer progression by modulating collagen cross-linking and maturation (Kurozumi et al., 2016). Wang et al. (2020) indicated that *PLOD2* can increase the resistance of GC cells to 5-fluorouracil by upregulating *BCRP* and inhibiting apoptosis. Several studies have indicated that *PLOD2* is correlated with poor prognosis of multiple cancers, including sarcoma, GC, lung cancer, renal cell carcinoma, breast cancer, cervical cancer, and bladder cancer (Miyamoto et al., 2016; Du et al., 2017; Kiyozumi et al., 2018; Chang et al., 2019). *PLOD2* as a potential regulator of peritoneal dissemination in GC (Kiyozumi et al., 2018). Regulation of the collagen cross-linking enzymes *LOXL2* and *PLOD2* by tumor-suppressive microRNA-26a/b in renal cell carcinoma. A feedback loop between hypoxia and matrix stress relaxation increases oxygen-Axis migration and metastasis in sarcoma (Chang et al., 2019). In this study, high-risk groups corresponding to shorter OS also had high levels of *PLOD2*.

*PHYHIP* is a protein-coding gene. That located on the p-arm of chromosome 8. Losses of the p-arm of chromosome 8 are frequently observed in breast cancer and other cancers. The research of Fumiichiro Yamamoto and Miyako Yamamoto shows that The expression of *PHYHIP* in breast cancer cell lines and clinical cases is down-regulated, which may be related to the occurrence and development of breast cancer (Yamamoto and Yamamoto, 2008).

*HPR* gene encodes a haptoglobin-related protein. This protein may be a clinically important predictor of recurrence of breast cancer (Kuhajda et al., 1989), Epelbaum R et al. found that *HPR* is a new tumor marker, which has potential use in the clinical setting of lymphoma (Epelbaum et al., 1998). Autophagy is the major intracellular degradation system, plays a fundamental role in cell, tissue, and organism homeostasis (Mizushima and Komatsu, 2011). Moreover, the role of these autophagy proteins in non-autophagy pathways are also emerging in many different biological contexts (Ktistakis and Tooze, 2016).

However, some limitations need to be considered in this study. Firstly, the data of this study was retrospective, which should be verified in prospective studies and multi-center clinical trials. Secondly, we were unable to investigate several key clinical features, including the definite pathological type of the tumors. Therefore, we cannot explore the relationship between autophagy genes and specific pathological GC types. Moreover, the present study only focused on ARGs, and the result could not represent all gene spectrum associated with GC. We will perform further experimental research *in vitro* and *in vivo* to investigate the precise functions and mechanisms of these genes in the regulation of autophagy-mediated tumorigenesis in GC.

## Conclusion

In this study, a risk score model based on a new set of six ARGs was identified by employing Lasso and Cox regression analyses on the data from TCGA-STAD dataset and GEO database. This model could be regarded as a better predictor of GC prognosis based on survival and diagnostic performance analyses, and as an independent predictor of the OS of GC patients. Meanwhile, this model – as a poor prognostic indicator – was positively associated with stromal and immune infiltration in GC, and with the PI3K/Akt and focal adhesive signaling pathways, which might promote the invasion and metastasis of GC cells.

## Data Availability Statement

Publicly available datasets were analyzed in this study. Patients with GC were downloaded from TCGA-STAD gene expression data in the GDC dataset (https://xenabrowser.net/). The gene expression profiles and clinical data of patients with GC were obtained from the GEO microarray dataset (GSE62254) as the validation cohort data. The data were analyzed using R software (https://www.r-project.org/). Human Autophagy Database (HADb) (http://autophagy.lu/clustering/index.html) and gene ontology (GO) enrichment-related autophagy genes (GO_AUTOPHAGY) Molecular Signatures Database (MSigDB v6.2, http://software.broadinstitute.org/gsea/msigdb).

## Author Contributions

JL analyzed the data. JL, KP, and CL contributed materials or analysis tools. KP prepared the figures and tables. JL and KP authored or reviewed drafts of the manuscript. YZ and YW conceived and designed the study. YZ revised the manuscript. All authors contributed to the article and approved the submitted version.

### Conflict of Interest

The authors declare that the research was conducted in the absence of any commercial or financial relationships that could be construed as a potential conflict of interest.

## Footnotes

^1^https://xenabrowser.net/

^2^https://www.r-project.org/

^3^http://autophagy.lu/clustering/index.html

^4^http://software.broadinstitute.org/gsea/msigdb

## Supplementary Material

Supplementary Table 1 List of 531 genes.

Supplementary Figure 1 Venn picture of the information of 531 ARGs from HADb (*n* = 232) and MSigDB (*n* = 394).

Supplementary Figure 2 (A) Disease-free interval (B) Disease-specific survival (C) Disease-free survival. The log-rank tests indicated that patients with high-risk score had worse prognosis compared with those with low-risk score (*p* = 0.05).
