# Supplementary figures and images for: A Novel Six-Gene-Based Prognostic Model Predicts Survival and Clinical Risk Score for Gastric Cancer
Source: Front Genet. 2021 Feb 22;12:615834. doi: 10.3389/fgene.2021.615834 (PMC7938863; doi:10.3389/fgene.2021.615834)

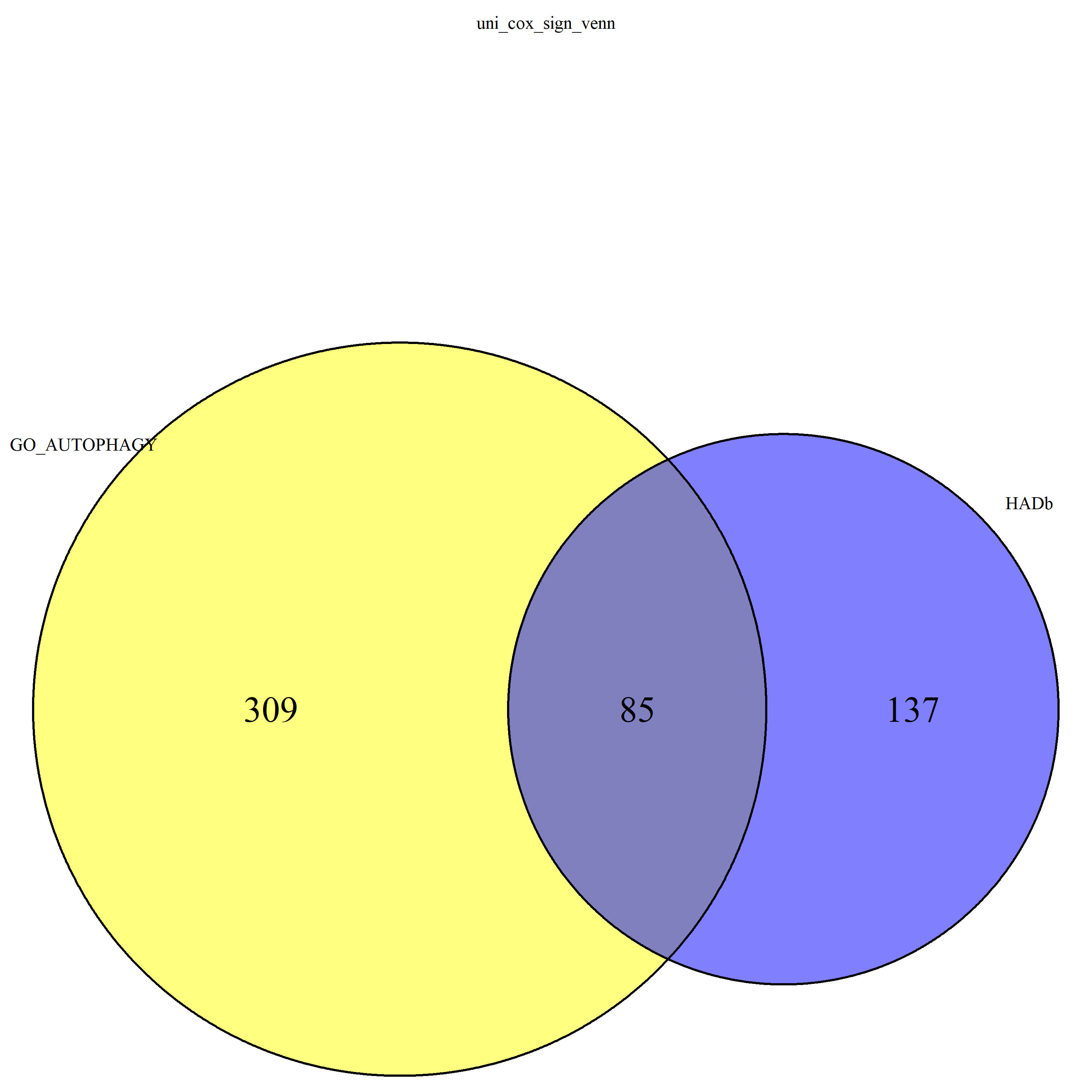

Supplement: Supplementary Figure 1 — Venn picture of the information of 531 ARGs from HADb (n = 232) and MSigDB (n = 394). [file Image_1.tiff]

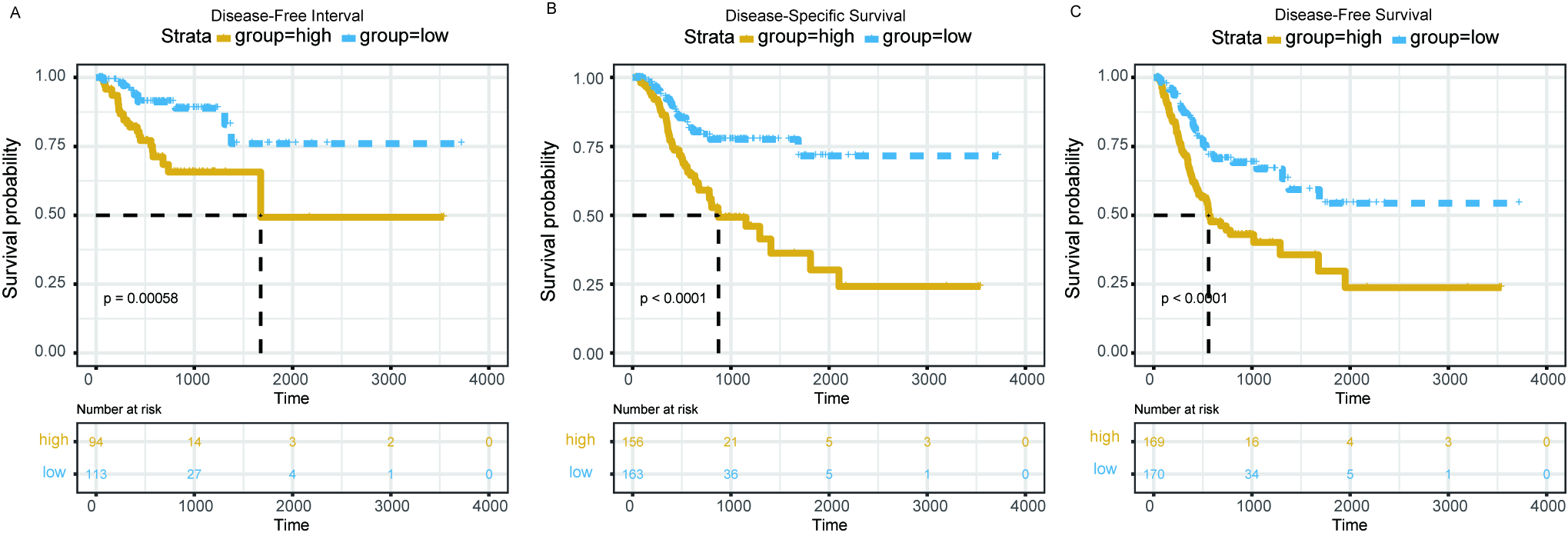

Supplement: Supplementary Figure 2 — (A) Disease-free interval (B) Disease-specific survival (C) Disease-free survival. The log-rank tests indicated that patients with high-risk score had worse prognosis compared with those with low-risk score (p = 0.05). [file Image_2.tif]
