# Supplementary material for: A Novel Six-Gene-Based Prognostic Model Predicts Survival and Clinical Risk Score for Gastric Cancer
Source: Front Genet. 2021 Feb 22;12:615834. doi: 10.3389/fgene.2021.615834 (PMC7938863; doi:10.3389/fgene.2021.615834)
Supplement: Supplementary file 4 [file Data_Sheet_1.pdf]

# Estimated age-standardized incidence rates (World) in 2020, stomach, both sexes, all ages

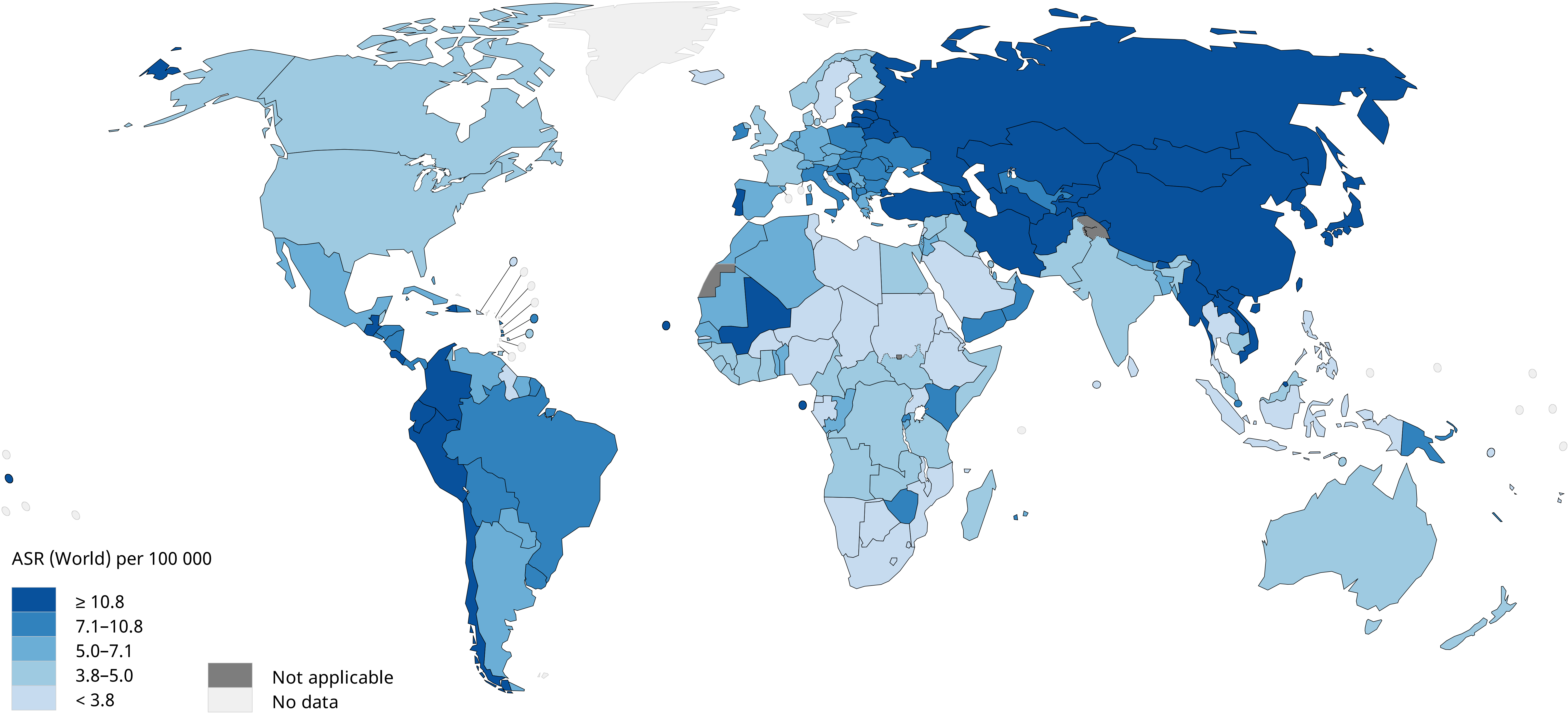


All rights reserved. The designations employed and the presentation of the material in this publication do not imply the expression of any opinion whatsoever on the part of the World Health Organization / International Agency for Research on Cancer concerning the legal status of any country, territory, city or area or of its authorities, or concerning the delimitation of its frontiers or boundaries. Dotted and dashed lines on maps represent approximate borderlines for which there may not yet be full agreement.

Data source: GLOBOCAN 2020
Graph production: IARC
(http://gco.iarc.fr/today)
World Health Organization

World Health Organization

© International Agency for Research on Cancer 2020
